# Hypersensitivity of Airway Reflexes Induced by Hydrogen Sulfide: Role of TRPA1 Receptors

**DOI:** 10.3390/ijms21113929

**Published:** 2020-05-30

**Authors:** Chi-Li Chung, You Shuei Lin, Nai-Ju Chan, Yueh-Yin Chen, Chun-Chun Hsu

**Affiliations:** 1 School of Respiratory Therapy, College of Medicine, Taipei Medical University, Taipei 110, Taiwan; clchung@tmu.edu.tw; 2 Division of Pulmonary Medicine, Department of Internal Medicine, Taipei Medical University Hospital, Taipei 110, Taiwan; 3 Division of Pulmonary Medicine, Department of Internal Medicine, School of Medicine, College of Medicine, Taipei Medical University, Taipei 110, Taiwan; 4 Department of Physiology, School of Medicine, College of Medicine, Taipei Medical University, Taipei 110, Taiwan; yslin@tmu.edu.tw; 5 Graduate Institute of Medical Sciences, College of Medicine, Taipei Medical University, Taipei 110, Taiwan; d119108008@tmu.edu.tw (N.-J.C.); d119108007@tmu.edu.tw (Y.-Y.C.)

**Keywords:** airway inflammation, airway reflex, C-fibers, H_2_S, TRPA1, sensory neuron

## Abstract

The activation of capsaicin-sensitive lung vagal (CSLV) afferents can elicit airway reflexes. Hypersensitivity of these afferents is known to contribute to the airway hypersensitivity during airway inflammation. Hydrogen sulfide (H_2_S) has been suggested as a potential therapeutic agent for airway hypersensitivity diseases, such as asthma, because of its relaxing effect on airway smooth muscle and anti-inflammatory effect. However, it is still unknown whether H_2_S affects airway reflexes. Our previous study demonstrated that exogenous application of H_2_S sensitized CSLV afferents and enhanced Ca^2+^ transients in CSLV neurons. The present study aimed to determine whether the H_2_S-induced sensitization leads to functional changes in airway reflexes and elevates the electrical excitability of the CSLV neurons. Our results showed that, first and foremost, in anesthetized, spontaneously breathing rats, the inhalation of aerosolized sodium hydrosulfide (NaHS, a donor of H_2_S; 5 mg/mL, 3 min) caused an enhancement in apneic response evoked by several stimulants of the CSLV afferents. This enhancement effect was found 5 min after NaHS inhalation and returned to control 30 min later. However, NaHS no longer enhanced the apneic response after perineural capsaicin treatment on both cervical vagi that blocked the conduction of CSLV fibers. Furthermore, the enhancing effect of NaHS on apneic response was totally abolished by pretreatment with intravenous HC-030031 (a TRPA1 antagonist; 8 mg/kg), whereas the potentiating effect was not affected by the pretreatment with the vehicle of HC-030031. We also found that intracerebroventricular infusion pretreated with HC-030031 failed to alter the potentiating effect of NaHS on the apneic response. Besides, the cough reflex elicited by capsaicin aerosol was enhanced by inhalation of NaHS in conscious guinea pigs. Nevertheless, this effect was entirely eliminated by pretreatment with HC-030031, not by its vehicle. Last but not least, voltage-clamp electrophysiological analysis of isolated rat CSLV neurons showed a similar pattern of potentiating effects of NaHS on capsaicin-induced inward current, and the involvement of TRPA1 receptors was also distinctly shown. In conclusion, these results suggest that H_2_S non-specifically enhances the airway reflex responses, at least in part, through action on the TRPA1 receptors expressed on the CSLV afferents. Therefore, H_2_S should be used with caution when applying for therapeutic purposes in airway hypersensitivity diseases.

## 1. Introduction

Hydrogen sulfide (H_2_S) is a colorless gas with an odor of rotten eggs and a potent inhaled irritant. The inhalation of H_2_S might lead to several adverse airway responses, such as cough, airway irritation, airway hypersensitivity, and lung inflammation [1,2]. Thus, H_2_S was recognized as an exogenous environmental toxin for decades. H_2_S is involved in a variety of functions, such as vasodilation [3], neuronal function regulation [4], inflammation [5], cellular signaling, and smooth muscle relaxation [6]. Moreover, endogenous H_2_S is primarily formed by cystathionine-β-synthase and cystathionine γ-lyase [7,8]. In 1996, Abe and Kimura first reported that, as an endogenous mediator, H_2_S regulates the neuronal function in CNS [7]. The H_2_S level is raised in serum and sputum in asthmatics, and the sputum H_2_S levels correlated inversely with pulmonary function [9]. H_2_S might play a role in the pathogenesis of asthma, and its level could be a biomarker of asthma. In addition, H_2_S was reported to cause relaxation in mouse bronchial smooth muscle [6]. Therefore, H_2_S was suggested as a treatment of asthma, although its role is still controversial.

The capsaicin-sensitive lung vagal (CSLV) afferents innervating the respiratory tracts are important in regulating respiratory functions under both normal and pathophysiological conditions [10,11,12]. The activation of these afferents can elicit various airway reflexes, including coughing and bronchoconstriction [10,11]. Thus, the hypersensitivity of CSLV afferents is believed to contribute to the airway hypersensitivity during airway inflammatory diseases, such as asthma and chronic cough [10,13,14].

In our previous study, we demonstrated that H_2_S induces hypersensitivity of CSLV afferents via TRPA1 receptors [15], which is a potent leading cause of airway hypersensitivity [16]. Airway hypersensitivity, characterized by hyper-responsive to airway sensory stimulus, is a key feature of asthma [14,17]. In words, on the one hand, the airway exposure of H_2_S might lead to relief of airway constriction [6]. On the other hand, it might induce airway hypersensitivity, such as chronic cough. While H_2_S increases the excitability of CSLV afferents [15], whether the enhanced sensory activities are able to trigger consequent airway reflex responses is still not known. To light up the information, the study was carried out to determine 1) whether the airway reflex response is enhanced after airway exposure to H_2_S in anesthetized, spontaneously breathing rats; if yes, 2) whether CSLV afferents play a role in the H_2_S-induced enhancement of airway reflex response; 3) whether H_2_S enhances cough reflex; 4) the role of TRPA1 receptors in the H_2_S-induced enhancement of airway reflex responses; and, 5) whether H_2_S directly elevates the excitability of isolated CSLV neurons via activation of TRPA1 receptors.

## 2. Results

### 2.1. In vivo Study

The animals were divided into 18 groups to conduct eight series of experiments. The *in vivo* study was carried out in 108 animals. Each group contained 6 animals.

**Figure 1:**
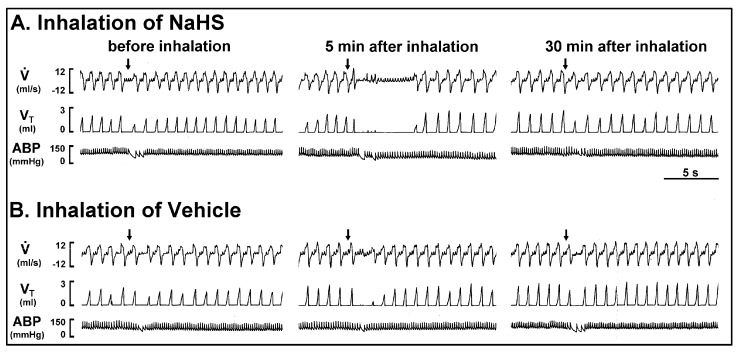
Experimental records illustrating the respiratory responses to a right-atrial bolus injection of capsaicin before, 5 min after, and 30 min after inhalation of sodium hydrosulfide (NaHS) (**A**) or its vehicle (**B**) in an anesthetized, spontaneously breathing rat (body weight: 320 g). NaHS (5 mg/mL) and its vehicle (isotonic saline) were delivered by aerosol inhalation for 3 min An elapsed time of 20 min was allowed between two inhalations. Right-atrial injections of capsaicin (1 µg/kg) are indicated by the arrows. V̇, respiratory flow; V_T_, tidal volume; ABP, arterial blood pressure. Please note that the apneic response elicited by the capsaicin injection was augmented by NaHS inhalation, but was not altered by vehicle inhalation.

**Figure 2:**
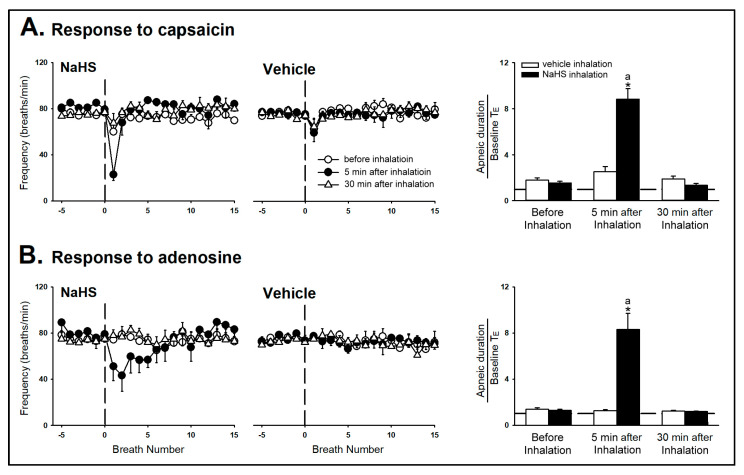
Effects of the inhalation of sodium hydrosulfide (NaHS) or its vehicle on the breathing frequency changes (left panel) and apneic response (right panel) to right-atrial injection of capsaicin (**A**) or adenosine (**B**) in two groups of anesthetized, spontaneously breathing rats. The apneic ratio was defined as the apneic duration occurring during 20 s after the capsaicin or adenosine injections divided by the baseline expiratory duration (T_E_). Data are means ± SE for six rats. ^a^, significantly different from before inhalation (*p* < 0.05); *, significant difference when corresponding data between vehicle and NaHS inhalation were compared (*p* < 0.05).

*Series 1 and 2.* In the control response, a right atrial bolus injection of capsaicin (1 μg/kg) elicited an inhibitory breathing pattern that was characterized by an apnea and reduced respiratory frequency. The apnea was revealed by several breaths with a mild prolongation of the expiratory duration (T_E_) (e.g., Figure 1). The apneic ratio was calculated by the apneic duration (longest T_E_) occurring during the first 20 s after the stimulant injections divided by the baseline T_E_ (apneic duration/baseline T_E_). Five minutes after the NaHS inhalation, the capsaicin-evoked respiratory inhibition responses were augmented (e.g., Figure 1 and Figure 2). The apneic ratio evoked by capsaicin significantly increased 5 min after NaHS inhalation (Figure 2A, before: 1.56 ± 0.14; 5 min after: 8.82 ± 0.92; *p* < 0.05), but not affected after vehicle inhalation (Figure 2A, before: 1.80 ± 0.20; 5 min after: 2.53 ± 0.45; *p* > 0.05). The reduced respiratory frequency caused by capsaicin was potentiated 5 min after NaHS inhalation as well. These augmented responses were not observed 30 min after termination of the NaHS inhalation (Figure 1 and Figure 2). The inhalation of NaHS or its vehicle had no detectable effect on the baseline mean arterial blood pressure and heart rate (Figure 1; Table 1). Additionally, the augmented effect of NaHS was also found in the respiratory inhibition that was evoked by adenosine (1.2 mg/kg) injection (Figure 2B, apneic ratio before NaHS: 1.28 ± 0.10; 5 min after NaHS: 8.30 ± 1.43; *p* < 0.05).

**Figure 3:**
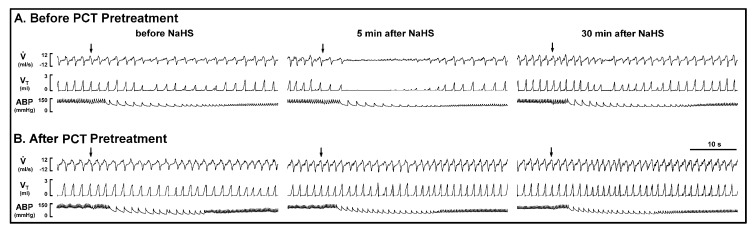
Experimental records illustrating the enhancing effect of sodium hydrosulfide (NaHS) inhalation on the apneic response evoked by adenosine injection (arrows, 1.2 mg/kg) before (**A**) and after (**B**) perineural capsaicin treatment (PCT) in an anesthetized, spontaneously breathing rat (body weight: 290 g). See legend of Figure 1 for further explanation. Note that the enhancing effect of NaHS inhalation on the apneic response evoked by adenosine injection was totally abolished after PCT pretreatment.

**Figure 4:**
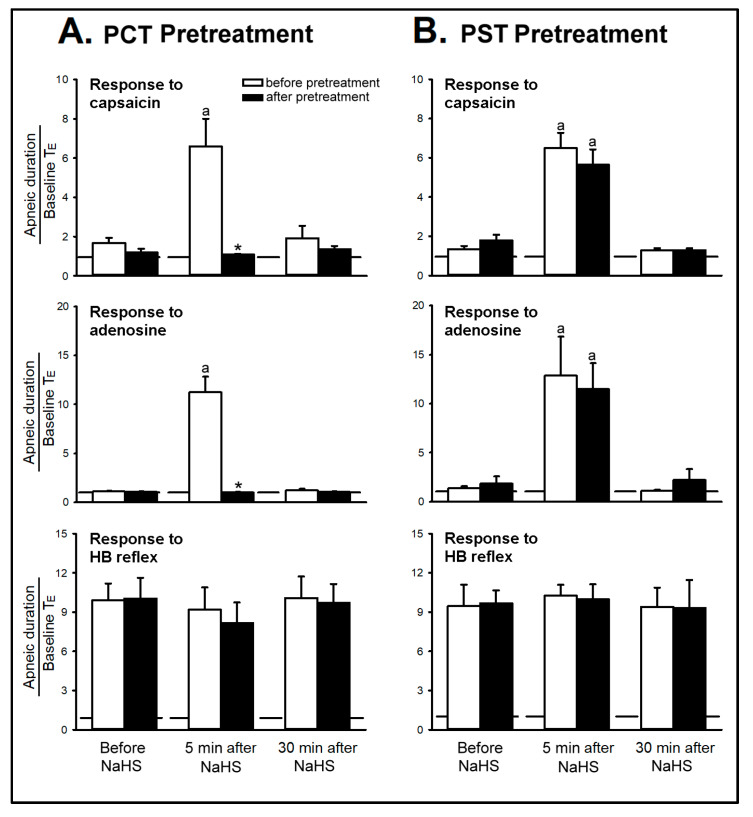
Effect of (**A**) perineural capsaicin treatment (PCT) and (**B**) perineural sham treatment (PST) on the enhancing effects of sodium hydrosulfide (NaHS) inhalation on apneic responses to right-atrial injections of capsaicin (1 µg/kg), adenosine (1.2 mg/kg) and Hering–Breuer (HB) reflex in six groups of anesthetized, spontaneously breathing rats. Data are means ± SE of six rats. ^a^, significantly different from before NaHS (*p* < 0.05); *, significant difference when corresponding data between before and after pretreatment were compared (*p* < 0.05).

*Series 3 and 4.* The CSLV afferents were blocked by the perineural capsaicin treatment (PCT) of the cervical vagi, a method modified from Jancso and Such [18], and the efficiency and selectivity of the PCT were assessed by capsaicin injection and Hering–Breuer (HB) reflex, respectively [18,19,20]. The HB reflex is a reflex initiated by lung inflation, which excited the myelinated fibers of vagus nerve, pulmonary stretch receptors [11,19]. In the present study, it showed that PCT abolished the adenosine-induced apnea (Figure 3) but did not affect the HB reflex (Figure 4), which suggested its selectivity on non-myelinated CSLV fibers. Furthermore, the apneic response evoked by capsaicin/adenosine and the sensitizing effect of the NaHS inhalation were both prevented by selectively blocking CSLV afferents with PCT (Figure 3B, Figure 4A), but not by perineural sham treatment (PST) (Figure 4B): the sensitizing effects of NaHS on the apneic ratio evoked by capsaicin were 6.61± 1.40 before PCT and 1.09 ± 0.03 after PCT (upper panel of Figure 4A, *p* < 0.05); the sensitizing effects of NaHS on the apneic ratio evoked by adenosine was 11.22 ± 1.68 before PCT and 1.03 ± 0.03 after PCT (middle panel of Figure 4A, *p* < 0.05). However, neither the NaHS, the PCT, nor the PST altered the HB reflex-induced apnea (lower panel of Figure 4).

**Figure 5:**
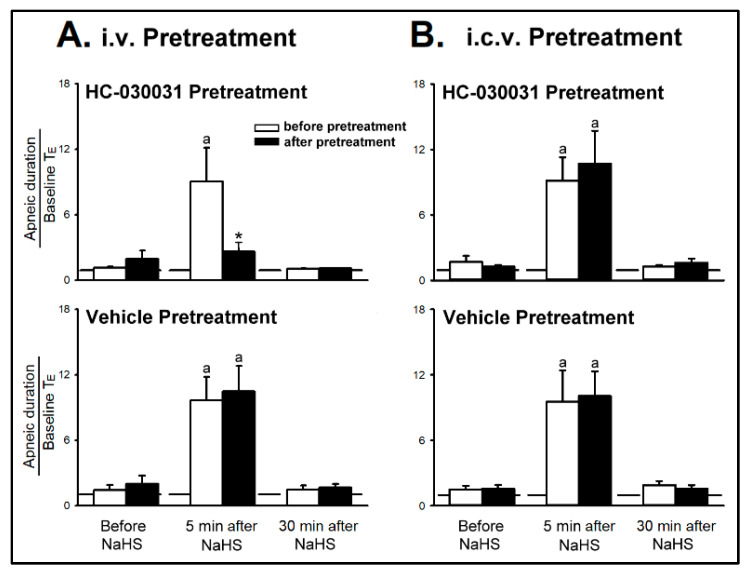
The role of TRPA1 receptors in sodium hydrosulfide (NaHS)-induced enhancement of apneic response in four groups of anesthetized, spontaneously breathing rats. (**A**) Effect of pretreatment of intravenous (i.v.) and (**B**) intracerebroventricular (i.c.v.) HC-030031 on the enhancing effects of NaHS inhalation on the apneic responses to right-atrial injections of adenosine (1.2 mg/kg). Data are means ± SE of six rats. ^a^, significantly different from before NaHS (*p* < 0.05); *, significant difference when corresponding data between before and after pretreatment were compared (*p* < 0.05).

*Series 5 and 6.* Remarkably, pretreatment with intravenous (iv) HC-030031 (8 mg/kg), but not its vehicle, blocked the augmenting effect of the NaHS inhalation on these apneic responses (Figure 5A). The NaHS-induced augmenting effects on the apneic response to adenosine was prevented by HC-030031 pretreatment (upper panel of Figure 5A, apneic ratio before and after the intravenous HC-030031= 9.06 ± 3.09 and 2.60 ± 0.80; *p* < 0.05), but not by vehicle pretreatment (lower panel of Figure 5A, apneic ratio before and after the vehicle pretreatment = 9.67 ± 2.08 and 10.50 ± 2.30; *p* > 0.05). Furthermore, pretreatment with HC-030031 by intracerebroventricular (icv) injection (1 μg/kg) did not alter the NaHS-induced potentiation in the apneic response to adenosine (Figure 5B, apneic ratio before and after the icv HC-030031 = 9.15 ± 2.13 and 10.69 ± 3.04; *p* > 0.05).

**Figure 6:**
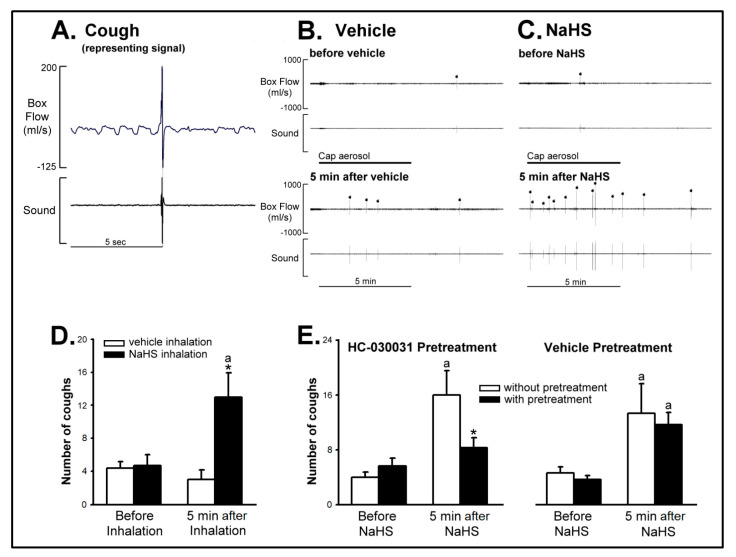
The role of TRPA1 receptors in sodium hydrosulfide (NaHS)-induced enhancement of cough reflex in six groups of awake guinea pigs. (**A**) Experimental record representing a signal of the cough reflex. Vertical bars show the flow changes and the sound signal in the exposure chamber. (**B**,**C**) Experimental records illustrating the effects of aerosolized vehicle and NaHS on cough response to capsaicin aerosol (30 μM, 5 min) in two guinea pigs. The duration of capsaicin aerosol delivery is indicated by horizontal bars. Each cough was indicated by the dot on the top. Data are expressed as the total number of coughs observed during a 5-min exposure period and the subsequent 5-min observation period after the end of capsaicin aerosol exposure. Each animal only received two capsaicin-induced cough challenges which were performed seven days apart to avoid tachyphylaxis. (**D**) Effects of aerosolized NaHS on cough responses to capsaicin before and 5 min after NaHS inhalation in two groups of awake guinea pigs. Data are means ± SE of six rats. ^a^, significantly different from before inhalation (*p* < 0.05); *, significant difference when corresponding data between vehicle and NaHS inhalation were compared (*p* < 0.05). (**E**) Role of TRPA1 receptors in NaHS-induced enhancement of cough reflex in four groups of awake guinea pigs. Data are means ± SE of six rats. ^a^, significantly different from before NaHS (*p* < 0.05); *, significant difference when corresponding data between without and with pretreatment were compared (*p* < 0.05). Please note that NaHS-induced enhancement of cough reflex was largely attenuated when guinea pigs were pretreated with HC-030031.

*Series 7 and 8.* Capsaicin aerosol (30 μM, 5 min) induced coughs in conscious, spontaneously breathing guinea pigs. The cough response was expressed as the total number of coughs observed during a 5-min exposure period and the subsequent 5-min observation period after the end of capsaicin aerosol exposure. The cough responses that were induced by capsaicin aerosol were potentiated in the NaHS inhalation group (Figure 6C,D, the number of coughs before and 5 min after NaHS: 2.33 ± 0.67 and 6.50 ± 1.48; *p* < 0.05), but not in the vehicle group (Figure 6B,D). Moreover, the NaHS-induced potentiating effect on cough reflex was almost abolished in the group of intraperitoneal (ip) HC-030031 (20 mg/kg) pretreatment (Figure 6E, the NaHS-enhanced cough responses without and with HC-030031: 8.00 ± 1.77 and 4.17 ± 0.70; *p* < 0.05). The vehicle of HC-030031 failed to do so (Figure 6E, the NaHS-enhanced cough responses without and with the vehicle of HC-030031: 6.67 ± 2.25 and 5.83 ± 0.91; *p* > 0.05).

### 2.2. In vitro study

The whole-cell perforated patch-clamp recording was performed in 30 CSLV neurons that were isolated from nodose and jugular ganglia of eight rats (weighing 50~150 g) and identified by the DiI fluorescence. The whole-cell capacitance of these neurons was 18.9 ± 1.1 pF (*n* = 30). To normalize the responses between neurons of different sizes, the current density (current/capacitance; pA/pF) was calculated for comparison.

**Figure 7:**
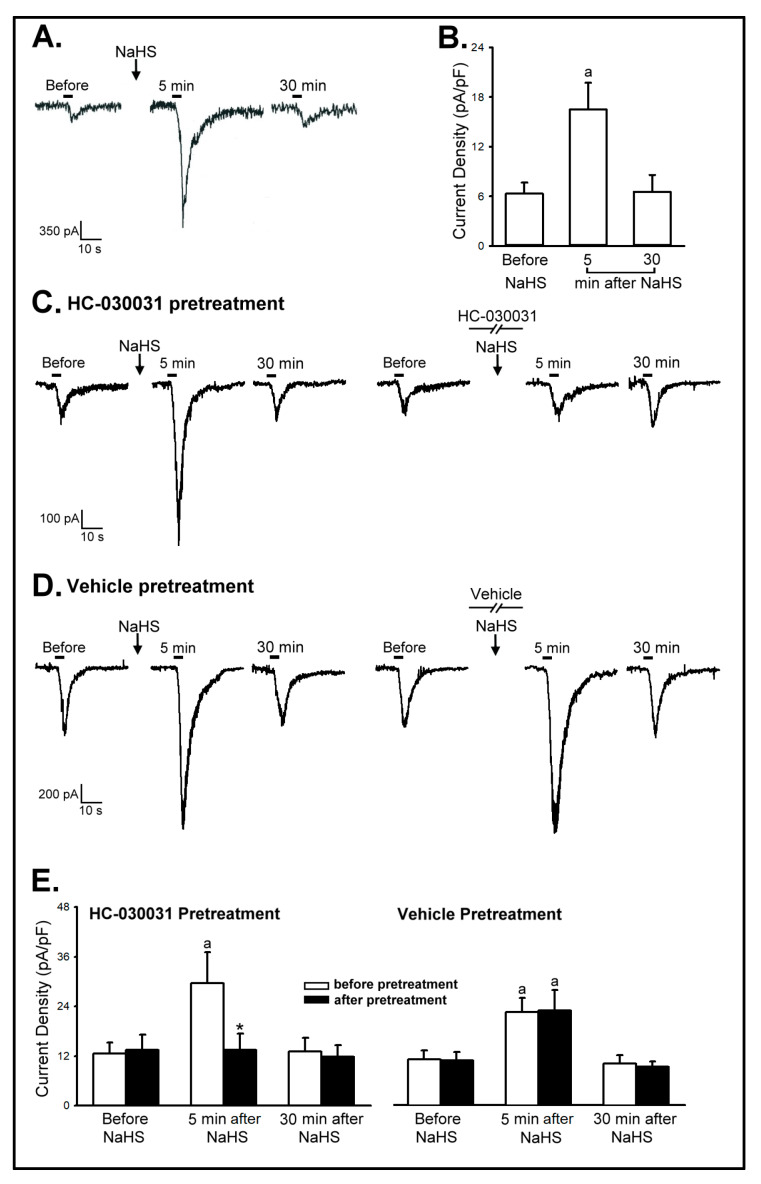
The role of TRPA1 receptors in sodium hydrosulfide (NaHS)-induced potentiating effect on inward current elicited by capsaicin in isolated rat capsaicin-sensitive lung vagal (CSLV) neurons. (**A**) Experimental records illustrating the inward current evoked by capsaicin (0.1 μM, 4 s; horizontal bars) before, 5 min after and 30 min after pretreatment of NaHS (100 μM, 5 min) in a rat CSLV neuron (jugular, 11.5 pF). An interval of 20–25 min elapsed between 2 capsaicin challenges for recovery. (**B**) Effects of NaHS on the capsaicin-evoked inward current before, 5 min after, and 30 min after NaHS in rat CSLV neurons (*n* = 10). The current density (current/capacitance; pA/pF) was calculated for comparison to normalize the responses between neurons of different sizes. The responses were similar between nodose and jugular neurons, and their data were pooled. Data are means ± SE. ^a^, significantly different from before NaHS (*p* < 0.05). Note that the capsaicin-evoked inward current was enhanced at 5 min after NaHS pretreatment. (**C**) Experimental records illustrating the effect of pretreatment with HC-030031 (20 μM, 16.5 min) on the NaHS-induced potentiating effect on capsaicin-evoked inward current in a rat CSLV neuron (jugular, 18.4 pF). (**D**) Experimental records illustrating the effect of pretreatment with the vehicle of HC-030031 on the NaHS-induced potentiating effect on capsaicin-evoked inward current in a rat CSLV neuron (nodose, 20.9 pF). (**E**) Effects of pretreatment with HC-030031 (*n* = 10) or its vehicle (*n* = 10) on the NaHS-induced potentiating effect on capsaicin-evoked inward current in rat CSLV neurons. Data are means ± SE. ^a^, significantly different from before NaHS (*p* < 0.05); *, significant difference when corresponding data between before and after pretreatment were compared (*p* < 0.05).

*Series 1.* The application of capsaicin (0.1 μM, 4 s) immediately evoked a small inward current in CSLV neurons (Figure 7A,B; 6.34 ± 1.34 pA/pF). However, the application of NaHS (100 μM, 5 min) markedly amplified the inward current responses to capsaicin. The current density was elevated to 16.49 ± 3.24 pA/pF (Figure 7A,B; *p* < 0.05; *n* = 10 from three rats). This potentiating effect of NaHS was returned to control when the capsaicin application was repeated 30 min later (Figure 7A,B; 6.55 ± 2.06 pA/pF).

*Series 2.* The potentiating effect of NaHS on current density was totally abolished by the HC-030031 (20 μM, 16.5 min) pretreatment (e.g., Figure 7C); after the HC-030031 pretreatment, the current density induced by capsaicin at 5 min after NaHS was 13.52 ± 3.90 pA/pF, which was not significantly different from the current density induced by capsaicin before NaHS (13.49 ± 3.69 pA/pF; *p* > 0.05; *n* = 10 from three rats), and clearly smaller than the response to capsaicin at 5 min after NaHS in the group before pretreatment of HC-030031 (Figure 7C,E; 29.54 ± 7.76 pA/pF; *p* < 0.05). In contrast, the pretreatment of the vehicle of HC-030031 did not affect the potentiating effects of NaHS on the current density elicited by capsaicin (e.g., Figure 7D); the current densities that were induced by capsaicin before and after vehicle pretreatment were 22.58 ± 3.27 pA/pF and 22.97 ± 4.60 pA/pF, respectively (Figure 7D,E; *p* > 0.05; *n* = 10 from two rats).

## 3. Discussion

This study demonstrated that the inhalation of NaHS (a donor of H_2_S) potentiated the apneic responses to capsaicin and to adenosine injections in anesthetized, spontaneously breathing rats. The sensitizing effects of NaHS were also found in cough reflex responses to aerosolized capsaicin in awake, spontaneously breathing guinea pigs. The inhalation of NaHS did not cause detectable cough reflex in the baseline. However, the cough responses to the capsaicin aerosol were significantly potentiated. Besides, pretreatment with HC-030031 abrogated the NaHS-evoked sensitizing effects on the apnea and cough responses, but was not affected by pretreatment with the vehicle of HC-030031. Furthermore, icv HC-030031 infusion did not alter the NaHS-evoked sensitizing effects on the apneic response. These results suggest that NaHS-induced sensitizing effects on apneic responses not through acting on CNS. In isolated rat CSLV neurons, this study demonstrated that NaHS potentiated the inward current evoked by capsaicin. Besides, NaHS also potentiated the Ca^2+^ transients elicited by adenosine (Figure S1). Consistent with our results in the *in vivo* experiments, the pretreatment of HC-030031 totally reversed the NaHS-induced potentiation of capsaicin-elicited inward currents and adenosine-evoked Ca^2+^ transients (Figure S1). Thus, these findings suggest that H_2_S induces a nonspecific sensitizing effect on CSLV afferents-mediated airway reflexes, and activation of TRPA1 receptors mediates this effect.

The activation of CSLV afferents can exert airway reflexes, such as apnea (in intact animals), cough, bronchoconstriction, and mucus secretion [11,21]. Several studies have indicated that H_2_S has involved in the pathogenesis of airway neurogenic inflammation through acting on the CSLV afferents [22,23]. Furthermore, our previous study has shown that exogenously applied H_2_S elevated the excitability of the CSLV afferents response to several stimulants [15]. In this present study, H_2_S-induced potentiation of the capsaicin/adenosine-evoked apneic responses was abolished by blockade of CSLV afferents with PCT, suggesting the involvement of CSLV afferents. The effectiveness of the PCT was validated by the blockade of the apneic reflex elicited by capsaicin, a selective CSLV afferents activator (e.g., upper panel of Figure 4A), and the selectivity of the PCT on unmyelinated afferents (including CSLV afferents) was elucidated by the persistence of apneic response evoked by HB reflex (e.g., lower panel of Figure 4A), which is conducted by large-diameter myelinated fibers and mediated through the activation of pulmonary stretch receptors [10,18].

The mechanism by which H_2_S potentiates the airway reflexes (apnea and cough) and enhances the excitability of CSLV neurons is not known. The sensitizing effects of the H_2_S had recovered within 30 min after termination of the inhalation, which suggests that the mechanism can be quickly inactivated after the termination of the H_2_S inhalation. In the present study, H_2_S-induced sensitization is believed to mediate through the activation of TRPA1 receptors. The role of TRPA1 receptors was verified by pretreatment with HC-030031, a selective antagonist of TRPA1. The efficacy and selectivity were supported by our previous study, being demonstrated on artificially ventilated rats [15]. The adenosine-triggered apnea was blocked by PCT in the present study (Figure 3), suggesting that it was acting via the activation of CSLV afferents [24,25]. Adenosine also elicited cardiovascular reflexes, such as hypotension and bradycardia via primary modulation of the AV node and the activation of A2 receptors on the vessel [24,26,27]. In this study, the NaHS-enhanced apneic response evoked by adenosine injection was abrogated after HC-030031 pretreatment, whereas the cardiovascular reflexes still existed, implying the contribution of TRPA1 receptors on the CSLV afferents. The TRPA1 receptor is a non-selective cationic channel [28]. However, capsaicin activates TRPV1 receptors, and adenosine activates specific metabotropic receptors [24,25,29]. Thus, the activation of TRPA1 receptors by H_2_S-induced sensitization might produce a nonspecific increase in the electrical excitability of these CSLV afferents [15] or generate interaction with these receptors [30]. 

A previous study has been reported that H_2_S induced TRPA1 activation by acting on two cysteine residues located in the N-terminal internal domain in nociceptive sensory neurons [31]. Moreover, previous studies have shown that TRPA1 activation elicits the influx of cations (e.g., Ca^2+^, Na^+^), leads to the opening of voltage-gated cation channels, and then presumably enhances the excitability of nociceptive neurons. Such a mechanism can explain the results of our studies that NaHS application potentiated the excitability of CSLV afferents, the inward currents (Figure 7), and CSLV-mediated airway reflexes (Figure 1, Figure 6). On the contrary, it has been reported that TRPA1 activation produced an inhibition of voltage-gated Ca^2+^ and Na^+^ channels in sensory neurons, reducing action potential-dependent neurotransimitters release, and the inhibition of C fibers-evoked excitation. This controversy of the consequences of TRPA1 activation might be possible due to the agonist concentration, route of administration, the species used, and the concentration of [Ca^2+^]_i_ achieved. Further investigations are needed to explore the relative contributions of these mechanisms in the H_2_S-induced sensitization of airway reflexes. The TRPA1 receptor is primarily expressed on nociceptive neurons, including CSLV neurons [32]. In the present study NaHS was applied by aerosol inhalation, in order to reduce systemic effect of H_2_S,. However, the arterial blood pressure slightly decreased, but no significant difference after NaHS inhalation (Table 1). We cannot rule out the possibility that the enhancing effects of NaHS resulted from H_2_S acting on the TRPA1 receptors expressed on the CNS. Notably, in addition to the abundant expression on the nociceptive neurons, TRPA1 receptors are also found in hippocampus and they play a role in regulating neuron excitability [33,34]. Therefore, we conducted the role of TRPA1 receptors in the potentiation effects of NaHS on apnea reflex by iv injection and icv infusion of HC-030031 in this study. However, the HC-030031 pretreatment by icv infusion did not alter the enhancing effects of NaHS on the apneic response (Figure 5B), suggesting that TRPA1 expressed on the CNS was not involved in this effect. This could not be due to insufficient dosing of HC-030031 because previous studies have shown the significant effects of icv HC-030031 in rodents [35]. Besides, the NaHS-induced potentiating effects on the inward current in isolated rat CSLV neurons were entirely blocked by HC-030031 pretreatment (Figure 7C,E), which indicated that the TRPA1 receptors expressed on the CSLV neurons, at least in part, play a vital role in this H_2_S-induced potentiation.

In addition to acting as a selective TRPA1 antagonist, HC-030031 has been accused of several side effects, such as block of voltage-gated calcium channels and other unexplained effects apart from the inhibition of TRPA1 [36,37]. However, these unwanted unselective effects of HC-030031 were usually observed at a high dose/concentration, such as 100 mg/kg [36] and 50~100 μM [37]. In the present study, a relatively low dose/concentration (8 mg/kg for anesthetized rats and 20 μM for CSLV neurons) were chosen in order to reduce the risk of the off-target effects of HC-030031. Our previous report [15] and experiment results (Figure S2) indicated that both responses of CSLV-afferents and -neurons to capsaicin (an agonist of TRPV1 channel) were unaffected by HC-030031, which supports the notion of its selectivity at such low dose/concentration. However, H_2_S/NaHS has also been described to activate KATP channels [38] and Cav3.2 channels [39]. Thus, a possibility that the contribution of those channels to the sensitizing effect of H_2_S should be considered. In the present study, two TRPA1 antagonists, HC-030031 (Figure 7) or AP-18 (Figure S3), eliminated the NaHS-induced potentiating effects on CSLV neurons; the agonist of TRPA1 mimicked the potentiating effects of NaHS on CSLV neurons, demonstrating an essential role of TRPA1 receptors (Figure S4). However, H_2_S/NaHS induced sensitization/activation of TRPA1 has been described to depend on reactive cysteines in TRPA1 [31]. Besides, agonists that activate TRPA1 receptors by this mechanism also sensitize TRPV1 receptors [40]. Several reactive TRPA1 agonists have been described to sensitize/activate TRPV1 at higher concentrations [41]. Thus, it should be seriously considered the role of TRPV1 receptors in the NaHS-induced potentiation. Particularly, the functional interaction between TRPA1 and TRPV1 has been largely reported [42,43,44].

Cough is an important reflex with high clinical significance [45,46]. Conscious guinea pig is the most typical and useful model for studying the cough reflex [47,48,49]. The inhalation of selective stimulants (e.g., capsaicin) of CSLV afferents reproducibly triggers coughs in various species, including humans [46,47,48,50]. Indeed, these CSLV afferents are known to play a vital role in cough hypersensitivity in airway inflammatory diseases [11,49,51]. In the current study, NaHS aerosol did not elicit cough responses in the baseline. However, the inhalation of NaHS enhanced the cough reflex induced by capsaicin aerosol (Figure 6C). Furthermore, the enhanced cough reflex was blocked entirely by the HC-030031, which suggests the vital role of TRPA1 activation in the cough hypersensitivity induced by H_2_S. In addition to CSLV afferents, another candidate for causing cough is rapidly adapting receptor (RAR), which does not directly respond to capsaicin [49]. Therefore, we could not rule out the possibility of the involvement of RAR in this cough hypersensitivity induced by H_2_S.

The precise role of H_2_S in airway inflammatory diseases remains controversial. H_2_S acts as a proinflammatory mediator in lipopolysaccharide-induced sepsis models and patients with COPD [52,53]. In contrast, H_2_S might play a role in anti-inflammatory effects in asthma and COPD [54,55]. The reason of the dispute of H_2_S might be due to the concentration, the release rate of the exogenous donor, and the duration of presence [56,57]. Previous studies have shown that H_2_S caused systemic inflammation and airway inflammation via TRPV1-mediated neurogenic inflammation in sepsis [5,53] and lung inflammation [22,23] model, respectively. Therefore, the possible mechanism of the proinflammatory effect of H_2_S might be through the neuropeptide release such as substance P and calcitonin gene-related peptide should be considered [21,42]. A recent study performed in our laboratory demonstrated that H_2_S elevated the sensitivity of rat CSLV afferents and potentiated the intracellular Ca^2+^ transients evoked by capsaicin in isolated rat CSLV neurons [15]. However, whether the increase in Ca^2+^ influx was sufficient to initiate the inward currents or action potentials was not known. The results of this study demonstrated that, without a significant effect on the baseline current, exogenous H_2_S elicited a sensitizing effect on the inward current responses to capsaicin in CSLV neurons through the activation of TRPA1 receptors (Figure 7). Furthermore, H_2_S is oxidized to polysulfide [58,59], which is believed to be a more potent TRPA1 agonist than H_2_S [60,61]. It has been reported that polysulfides are possible H_2_S-derived signaling molecules that stimulate TRP channels in rat astrocytes [60] and mouse DRG neurons [61]. Thus, we cannot rule out the possibility that polysulfide acts as the intermediate species of H_2_S-induced sensitizing effects in the CSLV neurons and airway reflexes.

It is well documented that the CSLV afferents activation elicits several airway reflexes, such as cough, bronchoconstriction, and mucus secretion [11,14,29,49]. When these afferents are sensitized by certain inflammatory mediators released during lung inflammation, it might exaggerate these triggered airway reflex responses [11,14,29,49]. However, increased H_2_S production and CSLV afferents sensitization are both believed to contribute to the pathogenesis of various lung inflammatory responses. [11,17,52,62,63]. Even H_2_S also plays protective roles in airway diseases, so that it could be used for therapeutic purposes [54,55]. H_2_S must be used carefully, since it has “the good and the bad” actions [56,57]. The results obtained from the present study suggest that, during lung inflammation, H_2_S might be the endogenous activator that contributes, at least in part, to the pathogenesis of airway hypersensitivity.

## 4. Materials and Methods

The following procedures were performed following the recommendations found in the “Guide for the Care and Use of Laboratory Animals” published by the National Institutes of Health and they were approved by the Institutional Animal Care and Use Committee of Taipei Medical University (2013-0228; 30 December 2013).

### 4.1. In vivo Study

#### 4.1.1. General Preparation

Male SD rats (weighing 290–420 g) were initially anesthetized with an ip injection of α-chloralose (100 mg/kg) and urethane (500 mg/kg) dissolved in a borax solution (2%). Supplemental doses of these anesthetics were intravenously administrated to sustain the elimination of pain reflexes produced by pinching the rat’s tail throughout the experiment. For the application of anesthetics and pharmacological agents, the left jugular vein was cannulated, and a catheter was advanced until its tip was positioned near the right atrium. The right femoral artery was cannulated to measure the arterial blood pressure. Body temperature was maintained at ~36 °C throughout the experiment using a servo-controlled heating pad. At the end of the experiment, the animals were euthanized while using an intravenous injection of KCl.

#### 4.1.2. Airway Exposure to H_2_S

H_2_S is an unstable gas. H_2_S is given by sodium hydrosulfide (NaHS, a donor of H_2_S) to achieve a stabilized application. NaHS is delivered into the lower airway of rats by aerosol inhalation (5 mg/mL, 3 min duration).

#### 4.1.3. Measurement of Respiratory Responses

Animals breathe spontaneously via the tracheal cannula. The respiratory flow was measured with a heated pneumotachograph and a differential pressure transducer, and the signal was integrated to give tidal volume. Signals of tidal volume (V_T_), expiratory duration (T_E_), respiratory frequency (f), minute ventilation (V̇e), heart rate (HR), and arterial blood pressure (ABP) were recorded on a polygraph recorder (MP 30; BIOPAC Instrument, Goleta, CA, USA) and also analyzed by an on-line computer (TS-100; BioCybernetics, Taipei, Taiwan). Before each chemical injection, the lungs were hyperinflated (tracheal pressure > 10 cmH_2_O) in order to establish a constant volume history.

#### 4.1.4. Perineural Capsaicin Treatment (PCT) of Cervical Vagi

In order to investigate the role of CSLV afferents in H_2_S-induced enhancement of cardiorespiratory reflexes, the PCT is a useful tool to provide a selective and reversible blockade of the bilateral cervical CSLV afferents, since PCT induces a reversible local axonal block [18,23,49]. Cotton strips soaked in high capsaicin solution (250 μg/mL) or its vehicle (perineural sham treatment, PST) are wrapped around a 2–3 mm segment of the isolated cervical vagi for 30 min The criterion for a successful PCT is based on the abolition of the reflex responses induced by the right atrial injection of capsaicin (a specific stimulant of CSLV afferents) and the existence of the Hering–Breuer (HB) reflex [19,64].

#### 4.1.5. Lateral Ventricle Implanted and Intracerebroventricular Infusion

A steel cannula (28-gauge, 0.64 mm OD) was inserted into the right lateral ventricle and glued to the skull by dental acrylic. The steel cannula was connected via silicon tubing to an infusion pump (Kent Scientific; Torrington, CT, USA). Either HC-030031 (1 μg/kg) or its vehicle were administrated icv in a volume of 5 μL, and the infusions were made at the rate of 1 μL/min controlled by the infusion pump.

#### 4.1.6. Assessment of Cough Reflex in Guinea Pig Model

Conscious male guinea pigs were placed in a 2.5 L restrained plethysmography chamber. Cough reflexes were triggered by capsaicin aerosol (30 μM, 5 min) generated with a vibrating plate nebulizer (Aeroneb Pro; Aerogen, Ltd., Galway, Ireland). Coughs were detected in two ways, including an internal microphone and a pressure transducer, which was equipped with a plethysmography chamber. The signals from the microphone and transducer were relayed to a polygraph, which provided a record of the number of coughs. The physiological parameters were analyzed with a computer that was equipped with an analog-to-digital converter and software (TS-100; BioCybernetics, Taipei, Taiwan). Each animal only received two capsaicin-induced cough challenges which were performed seven days apart to avoid tachyphylaxis [65,66].

#### 4.1.7. Experimental Protocols

The H_2_S was delivered by its donor NaHS. In order to minimize the systemic effect, NaHS solution (5 mg/mL) was provided by aerosol generated through a vibrating plate nebulizer. The nebulizer was connected to the tracheal cannula. The particle sizes of the aerosol were 1~5 μm, and the delivered flow was ~0.083 mL/min under the experimental conditions. Table 2 shows various experimental interventions in the study groups tested (Table 2). Series 1: to investigate the enhancing effect of H_2_S on respiratory reflexes, the respiratory responses elicited by intravenous bolus injections of capsaicin (a selective stimulant of CSLV afferents, 1 μg/kg) were compared 15 min before, 5 min after, and 30 min after the termination of airway exposure to NaHS or its vehicle (saline) in a group of rats (group 1). Series 2: to determine whether the H_2_S-induced sensitizing effect on respiratory reflexes was limited to capsaicin as a stimulant, another stimulant was chosen [adenosine (1.2 mg/kg) (group 2)]. The protocol of this study series was the same as that of study series 1. Series 3: to investigate the role of CSLV afferents, the H_2_S-induced sensitizing effect on respiratory responses to capsaicin were compared before and after PCT (group 3) or perineural sham treatment (PST) (group 4). Series 4: to confirm the specificity of the PCT, two other stimulants were chosen [adenosine (1.2 mg/kg) (groups 5–6) and Hering–Breuer reflex (10 cmH_2_O) (groups 7–8)]. The protocol of this study series was the same as that of study series 3. Series 5: to investigate the role of TRPA1 receptors in the H_2_S-induced sensitizing effect on the respiratory responses. As a control, adenosine was injected 15 min before, 5 min after, and 30 min after the NaHS inhalation. Afterward, the experiments were repeated 15 min after pretreatment with HC-030031 (8 mg/kg; iv) (group 9) or its vehicle (group 10). Series 6: to verify whether the H_2_S-induced sensitizing effect on respiratory responses was acted on CNS, the HC-030031 pretreatment was replaced by intracerebroventricular (icv) infusion. Adenosine was injected 15 min before, 5 min after, and 30 min after the NaHS inhalation. Subsequently, the experiments were repeated 15 min after pretreatment with icv HC-030031 (group 11) or its vehicle (group 12). Series 7: to investigate the sensitizing effect of H_2_S on cough reflexes in conscious guinea pigs, the cough responses elicited by capsaicin inhalation (30 μM, 5 min) were compared before and 5 min after the termination of airway exposure to NaHS or its vehicle (group 13–14). Series 8: to evaluate the role of TRPA1 receptors in the H_2_S-induced sensitizing effect on cough responses. The sensitizing effect of H_2_S on cough responses was assessed before and 5 min after NaHS without or with HC-030031 pretreatment (20 mg/kg, ip) (group 15–16); it was also assessed before and 5 min after NaHS without or with vehicle pretreatment (group 17–18).

### 4.2. In vitro study

The following experiments were performed *in vitro* preparation to determine whether H_2_S exerts a sensitizing effect directly on the CSLV neurons.

#### 4.2.1. Identification of CSLV Neurons

Sensory neurons innervating the lungs and airways were identified by retrograde labeling from the lungs with the fluorescent tracer 1,1’-dioctadecyl-3,3,3’,3’-tetramethylindocarbocyanine perchlorate (DiI), as described previously [42]. Briefly, young male SD rats (50~150 g) were anesthetized by isoflurane (2% in O_2_) through a vaporizing machine (AM Bickford, New York City, NY, USA). The DiI (0.2 mg/mL; 0.05 mL; 1% ethanol concentration) was instilled into the lungs through a needle (30 gauge) that was inserted into the tracheal lumen, and the incision was then closed. The animals were kept undisturbed for 7–10 days until they were euthanized for the cell culture to allow the DiI to be transported toward the soma of CSLV neurons [42].

#### 4.2.2. Isolation and Culture of Nodose and Jugular Ganglion Neurons

The methodology was described in detail in our previous studies [42]. The DiI-labeled SD rats were anesthetized with isoflurane and then decapitated. The head was quickly immersed in an ice-cold DMEM/F-12 solution, followed by the extraction of the nodose and jugular ganglia. Each ganglion was desheathed, cut, placed into a mixture of type IV collagenase (0.04%) and dispase II (0.02%), and then incubated for 60 min in 5% CO_2_ in air at 37 °C. The ganglion suspension was centrifuged (150 g, 5 min), and the supernatant was aspirated. The pellet was then resuspended in a modified DMEM/F12 solution and gently triturated. The dispersed cell suspension was centrifuged (500 g, 8 min) through a layer of bovine serum albumin (15%) to separate the cells from the myelin debris. The pellets were resuspended in the modified DMEM/F12 solution plated onto poly-L-lysine-coated glass coverslips and were then incubated overnight (5% CO_2_ in air at 37 °C).

#### 4.2.3. Electrophysiological Recording

Patch-clamp recordings were made in a whole-cell perforated-patch configuration (gramicidin 50 μg/mL) while using Axopatch 200B/pCLAMP 10.2 (Axon Instruments, Union City, CA), as described previously [42]. Briefly, the recording was performed at room temperature in a small-volume (0.2 mL) perfusion chamber that was continuously perfused by gravity feed (VC-6; Warner Instruments, Hamden, CT, USA) with extracellular solution (ECS) at 1 mL/min The tip resistance of borosilicate glass was pulled by using a micropipette puller and fire polished to 1.5–3.0 MΩ. The membrane perforation by gramicidin required 30–60 min after making a gigaseal in our preparation. We monitored and ensured the gramicidin perforation developed and the access resistance stabilized. Only cells with stable access resistance (generally ≤ 10 MΩ) were used for analysis. In all experiments of patch recording, the series resistance compensation was not employed. The regular ECS contained the following (in mM): 136 NaCl, 10 HEPES, 10 glucose, 5.40 KCl, 1.80 CaCl_2_, 1 MgCl_2_, and 0.33 NaH_2_PO_4_; the pH was adjusted to 7.4 with NaOH, and osmolarity was adjusted to 300 mOsm with glucose. The intracellular solution contained (in mM) the following: 92 potassium gluconate, 40 KCl, 8 NaCl, 1 CaCl_2_, 0.5 MgCl_2_, 10 EGTA, and 10 HEPES; the pH was adjusted to 7.2 with KOH, and osmolarity was adjusted to 300 mosM with glucose. Chemical solutions were applied using a three-channel fast-stepping perfusion system (SF-77B; Warner Instruments, Hamden, CT, USA), with its tip positioned to ensure that the cell was fully within the stream of the perfusate. All of the experiments were performed under the voltage-clamp mode, and the resting membrane potential was held at −70 mV. The data were filtered at 5 kHz.

#### 4.2.4. Experimental Protocols

The neurons were selected from the cultured cells for analysis that met the following criteria: 1) a spherical shape with no neurite outgrowths, 2) activated by capsaicin (0.1 μM, 4 s), and 3) labeled with DiI fluorescence. A total of 30 neurons were studied in two separate series of experiments. Series 1: to examine the sensitizing effect of H_2_S on the neurons, inward current elicited by capsaicin (0.1 μM, 4 s) were determined before, 5 min after and 30 min after the NaHS perfusion (100 μM, 5 min). Series 2: to evaluate the role of the TRPA1 receptors, the NaHS-induced potentiation of capsaicin-evoked inward currents were determined after the pretreatment of HC-030031 (20 μM, 16.5 min). HC-030031 was applied 8.5 min before, during, and 3 min after NaHS perfusion.

### 4.3. Pharmacological Agents

In the *in vivo* study, a stock solution of capsaicin (250 μg/mL) was prepared in 1% Tween 80, 1% ethanol, and 98% saline; and, a stock solution of adenosine (75 mg/mL) was prepared in saline. The solutions of capsaicin and adenosine for injection at the desired concentrations were prepared daily by dilution with saline based on the animal’s body weight. A stock of HC-030031 (30 mg/mL) was dissolved in DMSO and then further diluted to a final concentration of 2 mg/mL with a vehicle (10% Tween 80, 10% ethanol, and 80% saline) before use. In the *in vitro* study, desired concentrations of the pharmacological agents were prepared in a similar manner, except that the ECS, instead of saline, was used as the vehicle.

### 4.4. Data Analysis

The data were analyzed with a one-way or two-way repeated-measures ANOVA, followed by a post hoc Newman–Keuls test, unless mentioned otherwise. A value of *p* < 0.05 was considered to be significant. All data are means ± SE.

## 5. Conclusions

Inhalation of H_2_S might cause airway sensory hypersensitivity, which seems mediated through a direct action on the TRPA1 receptors expressed on these neurons. Thus, H_2_S should be used with caution for therapeutic purposes.

## Figures and Tables

**Table 1:** Effects of inhalation of NaHS or its vehicle on the baseline of mean arterial blood pressure (MABP) and heart rate (HR) in anesthetized, spontaneously breathing rats.

	Vehicle of NaHS (*n* = 12)	NaHS (*n* = 72)
MABP, mmHg		
Before inhalation	113 ± 4	112 ± 2
5 min after inhalation	111 ± 7	109 ± 2
30 min after inhalation	114 ± 5	112 ± 2
HR, beats/min		
Before inhalation	354 ± 15	357 ± 6
5 min after inhalation	344 ± 17	342 ± 8
30 min after inhalation	332 ± 15	359 ± 7

Data (means ± SE) are values averaged over 10-s periods before, 5 min and 30 min after the termination of inhalation. *n* represents rat numbers. No statistical significance was found between any two groups.

**Table 2:** Summary of the various experimental interventions in the study groups.

Study	Response	Series	Group	Inhalation(*in vivo*) /Perfusion (*in vitro*)	Stimulant	Pretreatment
*In vivo*	Respiratory responses	1	1	NaHS or its vehicle	Capsaicin	−
		2	2	NaHS or its vehicle	Adenosine	−
		3	3	NaHS	Capsaicin	PCT
			4	NaHS	Capsaicin	PST
		4	5	NaHS	Adenosine	PCT
			6	NaHS	Adenosine	PST
			7	NaHS	HB reflex	PCT
			8	NaHS	HB reflex	PST
		5	9	NaHS	Adenosine	iv HC
			10	NaHS	Adenosine	iv vehicle of HC
		6	11	NaHS	Adenosine	icv HC
			12	NaHS	Adenosine	icv vehicle of HC
	Cough reflex	7	13	NaHS	Capsaicin	−
			14	vehicle	Capsaicin	−
		8	15	NaHS	Capsaicin	without ip HC
			16	NaHS	Capsaicin	with ip HC
			17	NaHS	Capsaicin	without ip vehicle of HC
			18	NaHS	Capsaicin	with ip vehicle of HC
*In vitro*	Inward current	1	1	NaHS	Capsaicin	−
		2	2	NaHS	Capsaicin	HC
			3	NaHS	Capsaicin	vehicle of HC

PCT, perineural capsaicin treatment; PST, perineural sham treatment; iv, intravenous; HC, HC-030031; icv, intracerebroventricular; ip, intraperitoneal.

## Supplementary Materials

Supplementary materials can be found at https://www.mdpi.com/1422-0067/21/11/3929/s1.
