# Supplementary material for: Hypersensitivity of Airway Reflexes Induced by Hydrogen Sulfide: Role of TRPA1 Receptors
Source: Int J Mol Sci. 2020 May 30;21(11):3929. doi: 10.3390/ijms21113929 (PMC7312894; doi:10.3390/ijms21113929)
Supplement: Supplementary file 1 [file ijms-21-03929-s001.pdf]

Supplementary Information

Supplementary Method

Measurement of Ca^{2+} Transients

Ca^{2+} transients were measured in CSLV neurons with a Leica digital fluorescence microscope (DM IL LED; Leica Microsystems, Wetzlar, Germany) and a digital CCD camera (Zyla 4.2 sCMOS; Andor Technology, Belfast, UK). Neurons were washed or maintained with an extracellular solution (ECS) in a small-volume (0.2 ml) perfusion chamber at room temperature. Neurons were loaded with 5 μM fura-2 AM (Molecular Probes; Eugene, OR, USA) for 30 min at 37°C in tissue culture medium and then rinsed with ECS and allowed to deesterify for at least 30 min before use. The recording chamber was perfused continuously with ECS or the test chemicals by a gravity-fed valve control system (VC-66CS; Warner Instruments, Hamden, CT, USA). Dual images (340- and 380-nm excitation, 510-nm emission) were collected, and pseudocolored ratiometric images were monitored during the experiments. The image signals were continually analyzed at 2-s intervals during the experiments by using the MetaFluor® software (Universal imaging; West Chester, PA, USA). An increase in 340/380 ratio [△Ratio (F340/F380)] was measured as the difference between the peak amplitude of Ca^{2+} transients (4-s average) and the 30-s average at baseline.

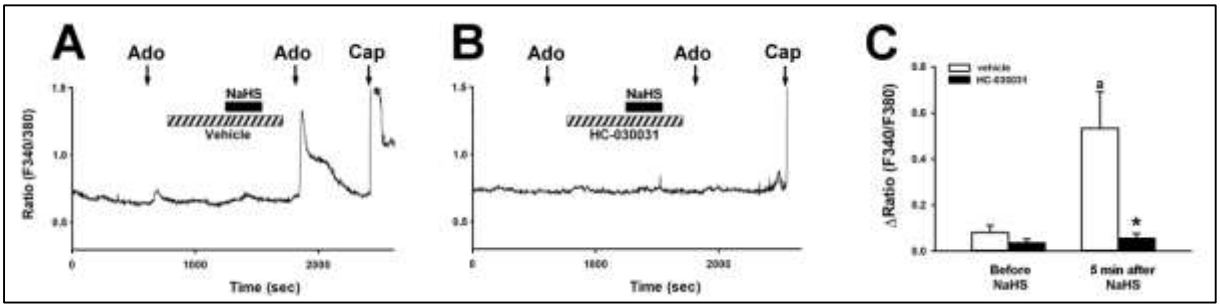

Figure S1. Effects of HC-030031 (20 μM, 16.5 min) on the NaHS (100 μM, 5 min)-induced potentiation of the Ca^{2+} transients evoked by adenosine in rat capsaicin-sensitive lung vagal (CSLV) neurons. **(A)** and **(B)**: experimental records illustrating the effects of pretreatments with vehicle or HC-030031, respectively, on the NaHS-potentiated Ca^{2+} transient evoked by adenosine (20 μM, 30 s). Adenosine was applied before and 5 min after the NaHS perfusion. Capsaicin (1 μM, 30 s) was applied to identify the CSLV neurons at the end of the both experiments. **(C):** group data showing HC-030031 (n=10), but not its vehicle (n=10), eliminated the NaHS-induced potentiating effects on the adenosine-evoked Ca^{2+} transients. Data are means ± SE. [a], significantly different from before NaHS ($P < 0.05$); *, significant difference when corresponding data between vehicle and HC-030031. Ado, adenosine; Cap, capsaicin.

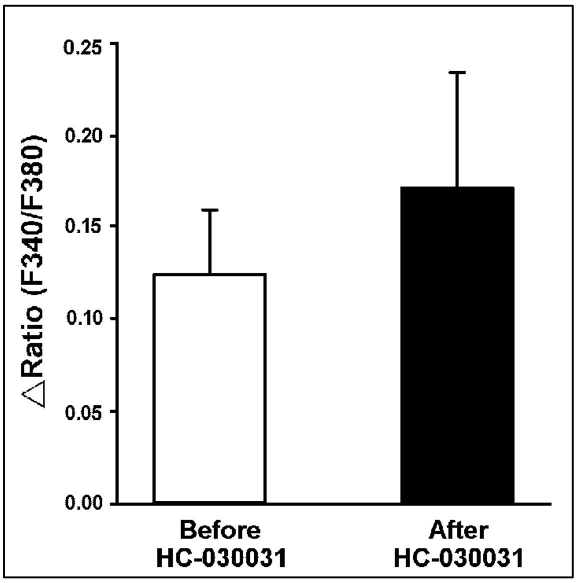

Figure S2. Effect of HC-030031 on capsaicin-induced Ca^{2+} transients in rat capsaicin-sensitive lung vagal (CSLV) neurons (n=10). Capsaicin (0.1 μM, 30 s)-evoked Ca^{2+} transients were compared before and after pretreatment of HC-030031 (20 μM, 16.5 min) or its vehicle. Time between 2 consecutive capsaicin challenges was 20 min. Data are means ± SE.

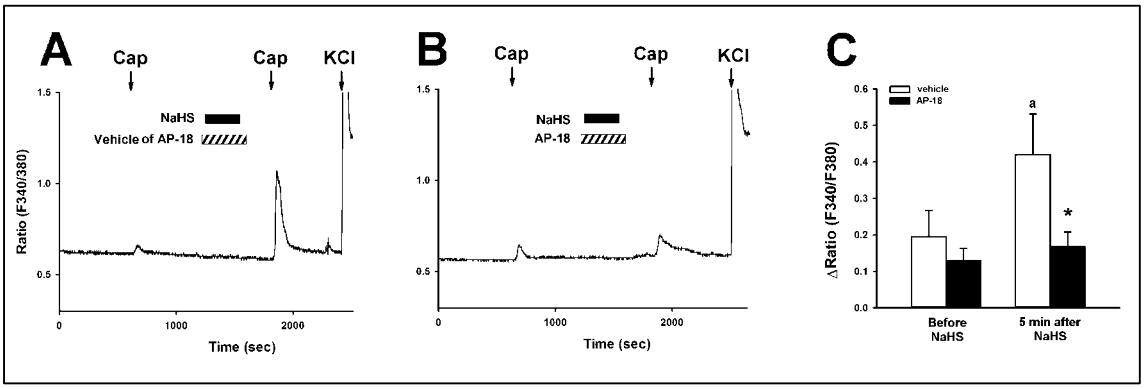

Figure S3. Effects of AP-18 (a TRPA1 antagonist; 40 μM, 6.5 min) on the NaHS (100 μM, 5 min)-induced potentiation of capsaicin-evoked Ca^{2+} transients in rat capsaicin-sensitive lung vagal (CSLV) neurons. **(A)** and **(B)**: experimental records illustrating the effects of pretreatments with vehicle or AP-18, respectively, on the NaHS-potentiated Ca^{2+} transient evoked by capsaicin (0.1 μM, 30 s). A KCl solution (60 mM, 30 s) was applied to test the cell vitality at the end of both experiments. **(C)**: group data showing AP-18 (n =15), but not its vehicle (n=20), eliminated the NaHS-induced potentiating effects on the capsaicin-evoked Ca^{2+} transients. Data are means ± SE. [a], significantly different from before NaHS ($P < 0.05$); *, significant difference when corresponding data between vehicle and AP-18. Cap, capsaicin.

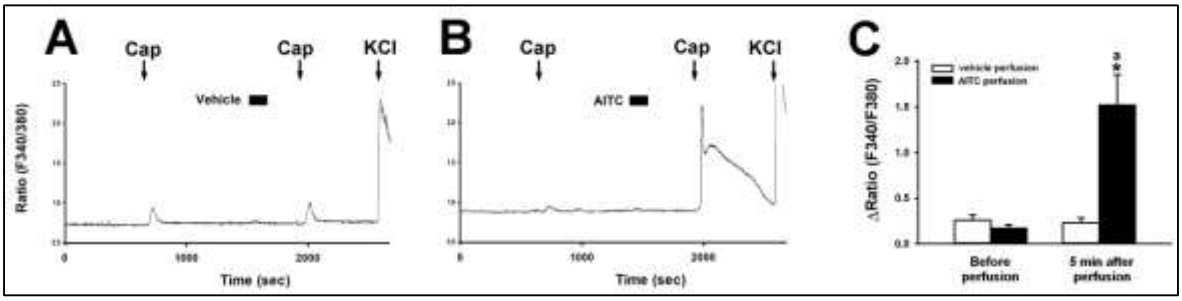

Figure S4. Allyl isothiocyanate (AITC; a TRPA1 agonist, 30 μM, 2.5 min)-induced potentiation of the Ca^{2+} transients evoked by capsaicin in rat capsaicin-sensitive lung vagal (CSLV) neurons. **(A)** and **(B)**: experimental records illustrating the effects of pretreatments with vehicle or AITC, respectively, on the capsaicin-induced Ca^{2+} transients. Capsaicin (0.1 μM, 30 s) was applied before and 5 min after the AITC or vehicle perfusion. A KCl solution (60 mM, 30 s) was applied to test the cell vitality at the end of both experiments. **(C):** group data showing AITC (n=22), but not its vehicle (n=22), potentiated the capsaicin-induced Ca^{2+} transients. Data are means ± SE. [a], significantly different from before perfusion ($P < 0.05$); *, significant difference when corresponding data between vehicle and AITC.
